# Application Usability, Engagement, and Postpartum Weight Retention: Secondary Analysis of the INTER-ACT Randomized Controlled Trial

**DOI:** 10.1016/j.mcpdig.2026.100381

**Published:** 2026-06-09

**Authors:** Lisanne Duizer, Femke Geusens, Emma Geerits, Roland Devlieger, Annick Bogaerts

**Affiliations:** a REALIFE Research Group, Research Unit Woman and Child, Department of Development and Regeneration, KU Leuven, Leuven, Belgium; b Department of Clinical Child and Family Studies, Vrije Universiteit Amsterdam, The Netherlands; c Department of Women’s and Children’s Health, Uppsala University, Uppsala, Sweden; d REVAL Rehabilitation Research Center, UHasselt, Diepenbeek, Belgium; e Department of Obstetrics and Gynecology, University Hospitals Leuven, Leuven, Belgium; f Department of Obstetrics, Gynecology and Reproduction, St-Augustinus Hospital, Wilrijk, Belgium; g Faculty of Health, University of Plymouth, Devon, United Kingdom

## Abstract

**Objective:**

To examine whether perceived usability of the INTER-ACT application influences postpartum weight loss and whether this effect is mediated by application use, motivational power, and implementation of lifestyle recommendations.

**Patients and Methods:**

Dutch-speaking women aged 18 years or older who had delivered a singleton infant and exceeded the recommended guidelines of the Institute of Medicine for gestational weight gain got recruited from May 2017 to April 2019, Across 6 Flemish hospitals. This secondary analysis included 138 participants of the INTER-ACT intervention arm. At 6 months postpartum participants completed a process evaluation survey, assessing application usability using the system usability scale, perceived motivational power, and perceived implementation of lifestyle recommendations. Weight loss was calculated between 6 weeks and 6 months postpartum. Pearson correlations and parallel mediation analyses were conducted, adjusting for maternal age, parity, and prepregnancy body mass index.

**Results:**

Application usability was moderate (mean system usability scale=60.6) and positively associated with perceived application use frequency (B=.05, *P*<.001), motivational power (B=.003, *P*<.001), and implementation of lifestyle recommendations (B=.01, *P*=.036), but not with weight loss. Usability showed no total, direct, or indirect effect on weight loss. Gestational weight gain (GWG) was the only significant predictor (B=0.24, *P*=.003). Higher usability was also associated with more positive and fewer negative emotional responses (*P*<.05).

**Conclusion:**

Application usability was associated with engagement and positive emotional experience, but not with reduced PPWR. The GWG remained the main determinant of postpartum weight outcomes. Preventing excessive GWG and complementing this with emotionally supportive postpartum mHealth tools may improve outcomes.

**Trial Registration:**

clinicaltrials.gov Identifier: NCT02989142

Postpartum weight retention (PPWR), defined as the difference between prepregnancy weight and weight retained after childbirth, is a key contributor to long-term maternal obesity. Excessive postpartum weight retention (EPPWR; 5 kgs retained at 6 months postpartum) increases the risk of future cardiometabolic conditions, and is associated with poor outcomes in subsequent pregnancies, such as gestational diabetes and hypertension.^1^, ^2^, ^3^, ^4^, ^5^, ^6^ Psychosocially, persistent PPWR is linked to lower self-esteem, postpartum depression, and body dissatisfaction.^7^^,^^8^ Despite these risks, losing pregnancy weight remains challenging as around 75% of women do not return to their prepregnancy weight within the first postpartum year.^9^ Given these clinical and psychological implications, reducing PPWR is a key priority in postpartum care.

The postpartum period involves considerable physiological, emotional, and logistical challenges, including recovery from childbirth, sleep disruption, and time scarcity, which complicate the adoption of healthy behaviors.^10^, ^11^, ^12^ Mobile health (mHealth) tools may help overcome these barriers by offering flexible, personalized, and on-demand lifestyle support.^13^^,^^14^

However, the effectiveness of mHealth interventions depends strongly on usability. According to the ISO 9241-11 framework, an international standard for evaluating human-system interaction, usability refers to how effectively, efficiently, and satisfactorily users can achieve intended goals within a specific context of use.^15^ This definition is particularly relevant for postpartum women, who frequently interact with applications under conditions of time pressure, fatigue, and emotional stress.^16^ Low usability can hinder task completion, evoke frustration, and ultimately contribute to disengagement. Conversely, well-designed applications may facilitate continued use, sustain motivation, and promote behavioral change.^17^^,^^18^

User experience (UX) extends beyond usability, encompassing emotional responses, aesthetics, engagement, and perceived usefulness.^15^^,^^19^ For postpartum women, this means that an application is not only evaluated based on its functionality but also on how it makes them feel during moments of stress or vulnerability. Usability is closely linked to emotional experience: poorly designed applications may evoke frustration, whereas intuitive applications can foster competence and satisfaction.^20^^,^^21^

Although mHealth interventions are increasingly used to support postpartum women, no studies to our knowledge have directly examined how application usability affects behavioral implementation and PPWR outcomes. Evidence from other intervention fields suggests that application usability can significantly affect user engagement and behavioral change.^22^^,^^23^ However, it remains unclear whether these findings translate to postpartum women, who face unique challenges in the early months after childbirth. Emerging evidence suggests that more frequent application engagement is associated with healthier eating behaviors and weight loss among postpartum women.^24^^,^^25^

On the basis of these insights, we hypothesize that usability is associated with behavioral outcomes. Specifically, if an application is perceived as easy to use and supportive, users may be more likely to improve their diet or physical activity, thereby reducing PPWR. Conversely, poor usability may hinder behavioral change regardless of content quality.

To address this gap, we examine the association between usability features of a postpartum mHealth tool and PPWR. The analysis is embedded within the INTER-ACT randomized controlled trial, which targets women with excessive gestational weight gain (EGWG), a group at elevated risk of PPWR.^26^ The study represents a prespecified secondary analysis of the INTER-ACT trial. Within this framework, we examine the engagement pathways linking usability to postpartum outcomes. Three mediators are included in the model to clarify the mechanisms through which usability affects PPWR: application use frequency, perceived motivational power, and perceived implementation of lifestyle recommendations. In doing so, this study aims to provide actionable insights for designing and evaluating mHealth tools in maternal healthcare, with a focus on usability, user experience, emotional engagement, and effective content delivery.

## Patients and Methods

### Study Design and Participants

This secondary analysis utilized data from the intervention arm of the INTERpregnAncy Coaching for a healthy future (INTER-ACT; ClinicalTrials.gov: NCT02989142, https://clinicaltrials.gov/study/NCT02989142) multi-center randomized controlled trial, conducted between May 2017 and April 2019 across 6 Flemish hospitals. Follow-up of participants continued until delivery of the subsequent pregnancy. The INTER-ACT trial tested whether a lifestyle intervention spanning the interpregnancy period and subsequent pregnancy reduced pregnancy complications, defined as a composite outcome of pregnancy-induced hypertension, gestational diabetes, cesarean section, and large-for-gestational-age, in women with prior . The primary trial found no relevant reduction in this composite outcome (odds ratio [OR] 1.29; 95% CI, 0.85-1.96; *P*=.23).^27^^,^^28^

This study presents a prespecified secondary analysis of the intervention arm. Eligible participants were Dutch-speaking women aged ≥18 years who delivered a singleton infant and exceeded the Institute of Medicine GWG guidelines.^29^ Exclusion criteria were chronic illnesses requiring dietary restrictions or medication, psychiatric conditions, or contraindications for physical activity.

**Figure 1:**
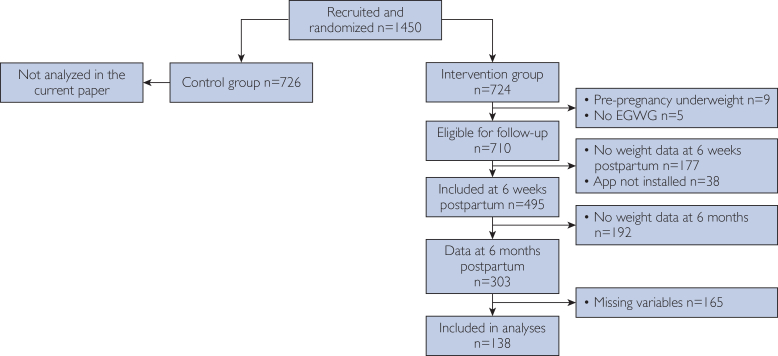
Flowchart of participant recruitment and final sample size. The flowchart illustrates the recruitment process for the INTER-ACT randomized controlled trial, detailing the number of participants assessed for eligibility, excluded, randomized to the intervention arm, and included in the final analytic sample (N=138) for this secondary analysis based on completion of the process evaluation survey and availability of weight data at 6 weeks and 6 months postpartum.

Only women in the intervention group with complete UX survey data and weight measurements at 6 weeks and 6 months postpartum were included (N=138) (Figure 1). Baseline characteristics (prepregnancy weight, GWG, and weight loss at 6 weeks and 6 months postpartum) of the included sample did not differ from those of the full intervention group.

### Intervention and Application Use

The INTER-ACT application was designed to support general healthy lifestyle behaviors across nutrition, physical activity, and mental well-being during the interpregnancy and pregnancy periods; postpartum weight loss was not a standalone primary target of the application.

The INTER-ACT intervention included a postpartum phase (6 weeks-6 months postpartum) and an interpregnancy phase; only the postpartum phase is analyzed here.

Participants received 4 structured, face-to-face lifestyle coaching sessions at approximately 6, 8, and 12 weeks, and at 6 months postpartum. Coaching addressed 3 behavioral domains: nutrition (with emphasis on eating behavior), physical activity (including sedentary behavior reduction), and mental well-being.

In addition to coaching, participants received access to the INTER-ACT application, designed to support behavior change between sessions. The application provided personalized motivational messages, practical information, health-related advice, and individually tailored tips related to nutrition, activity, and emotional health. Users could define specific goals within the application, track progress, and record goal achievements.

To facilitate self-monitoring, participants were provided with a bluetooth-connected weight scale and pedometer. Data from these devices were automatically synced with the application, allowing users to monitor their weight and physical activity levels and enabling coaches to provide personalized feedback during coaching sessions. In addition, participants also received periodic in-application questionnaires assessing lifestyle behaviors, well-being, engagement, and process evaluation. All application content was available exclusively in Dutch to ensure accessibility and ease of use for participants.

### Ethics

Informed consent was obtained from all participants. The study was conducted according to the Declaration of Helsinki and was approved by the clinical trial center/ethical committee UZ Leuven (protocol code B322201730956/S59889). The authors confirm that all ongoing and related trials for this intervention are registered.

### Measures

#### Perceived Application Use Frequency

Participants reported the number of days per week they used the application (0-7 days). This approach was chosen as it captures users perceived engagement with the intervention and was consistently available across participants. Although objective usage metrics (eg, log data) may provide more precise estimates of engagement, self-reported use was used to ensure conceptual consistency across measures, as other engagement indicators in the study were also based on self-reported perceptions.

#### Perceived Application Motivational Power

Participants completed 4 self-developed Dutch items assessing perceived application motivational power. The items were as follows: (1) the application motivated me to eat healthier, (2) the application motivated me to be more physically active, (3) the application helped me feel better about myself, and (4) the tracking of my weight changes on the application motivated me to adopt healthy lifestyle habits. Responses were provided on a 5-point Likert scale (1=strongly disagree, and 5= strongly agree).

#### Perceived Motivational Power and Implementation of Lifestyle Recommendations

Perceived application motivational power (4 items) and perceived implementation of lifestyle recommendations (3 items) were assessed using self-developed Dutch items on a 5-point Likert scale (1=strongly disagree and 5=strongly agree). Principal Component Analysis with Varimax rotation revealed two distinct factors corresponding to motivational power and implementation of lifestyle recommendations. Internal consistency was satisfactory (α=0.79 and α=0.89, respectively). The mean scores were calculated for each factor and used in further analyses.

#### Perceived Emotional Responses

Perceived emotional responses to application use were assessed using 6 self-developed Dutch items addressing emotions experienced during overall application use and in response to specific application components (e.g., lifestyle tips, step counts, and weight changes).

### System Usability Scale score

Perceived application usability was assessed using the system usability scale (SUS).^34^^,^^35^ The SUS consists of 10 items rated on a 5-point Likert scale. Scores were calculated according to standard guidelines, yielding a total score ranging from 0 to 100, with higher scores indicating better usability. Individual SUS scores were used in subsequent analyses. The SUS score questionnaire is provided in the Supplemental Appendix (available online at https://www.mcpdigitalhealth.org/).

### Weight Loss at 6 Months Postpartum

The primary outcome, PPWR, was calculated by subtracting the participant’s self-reported prepregnancy weight from the measured weight at 6 months postpartum, as recorded by trained study personnel. Prepregnancy weight was self-reported by participants at recruitment, which took place a few days after delivery at the time of informed consent signing. Self-reported prepregnancy weight is commonly used in pregnancy research, as objective preconception measurements are often unavailable, partly due to the high proportion of unplanned pregnancies.^6^^,^^30^ PPWR was analyzed both continuously (kg retained) and categorically, with substantial weight retention defined as ≥5 kilograms retained, a commonly used clinically meaningful indicator of long-term weight gain risk in postpartum research.^2^^,^^30^^,^^31^

Because participants-initiated application use at 6 weeks postpartum, weight loss was defined as the difference between weight at 6 weeks and weight at 6 months postpartum to isolate the potential impact of application engagement. Prepregnancy weight and weight at delivery were self-reported by participants. Both were used to calculate GWG and determine excessive GWG according to the National Academy of Medicine guidelines.

### Control Variables

Covariates included maternal age, prepregnancy BMI, and parity.^31^^,^^32^

### Statistical analysis

All analyses were conducted using IBM SPSS Statistics version 29, with mediation models estimated using the PROCESS macro v5.0.^33^ Descriptive statistics (means and SDs, percentages) were calculated for participant characteristics and key variables. Pearson correlations examined bivariate associations between usability (SUS score), perceived application use frequency, perceived application motivational power, and perceived implementation of lifestyle recommendations.

A parallel mediation model (PROCESS Model 4) was used to test whether the SUS scores were associated with weight loss at 6 months postpartum through 3 mediators, ie, perceived motivational power, perceived implementation of lifestyle recommendations, and perceived application use frequency. The model was adjusted for prepregnancy BMI, maternal age, and parity. Bias-corrected 95% CIs for indirect associations were estimated using 5000 bootstrap resamples, with associations considered relevant when CIs did not include zero.

To examine whether application usability was associated with participants perceived emotional response, a multivariate general linear model was conducted with SUS scores as the independent variable and the set of emotional response indicators as dependent variable.

## Results

### Participants Characteristics

Descriptive statistics for the sample (N=138) are presented in Table 1. Participants had a mean prepregnancy weight of 71.16 kg (SD=11.58). Weight loss averaged 11.01 kg (SD=3.50) at 6 weeks postpartum, with an additional 2.69 kg (SD=3.39) lost between 6 weeks and 6 months postpartum. The mean PPWR was 4.08 kg (SD=4.52), with 39.1% of participants exhibiting EPPWR.

**Table 1:** Participant Characteristics of the INTER-ACT Intervention Arm^a^

Participants characteristics	Min	Max	Mean ± SD
Weight (kg)			
Prepregnancy weight	47.0	105.0	71.16 ± 11.58
Weight loss at 6 wks postpartum	−5.60^b^	21.30	11.01 ± 3.50
Weight loss at 6 mo postpartum	−4.70^b^	12.70	2.69 ± 3.39
PPWR	−8.40^b^	18.30	4.08 ± 4.52
Total GWG	10.0	33.7	17.78 ± 4.14
Prepregnancy BMI	18.87	37.11	25.54 ± 4.10
Demographics			
Age	21	40	30.78 ± 3.85
Children	1	4	1.65 ± 0.79
Application days per wk			
Perceived application use frequency	0.00	7.00	3.85 ± 1.91
Perceived usability			
SUS score	15.00	100	60.60 ± 14.35
Multiparous, n %	47.8%		
Exclusively breastfeeding 6 wks postpartum, n %	70.3%		
EPPWR, n %	39.1%		
Emotional responses to application use, n %			
Happiness	38.3%		
Irritation	21.9%		
Curiosity	37.7%		
Sadness	5.6%		
Guilty	11.1%		
Insecurity	9.7%		
Surprised	8.2%		
Angry	2.8%		
Persuaded	15.9%		
Anxiety	0.5%		

a Abbreviations: BMI, body mass index; EPPWR, Excessive postpartum weight retention; GWG, gestational weight gain; PPWR, Postpartum weight retention; SUS, system usability scale.

b Negative values indicate weight gain.

Total GWG averaged 17.78 kg (SD=4.14), with participants exceeding the recommended Institute of Medicine weight gain guidelines by an average of 4.59 kg (SD=3.95). The mean prepregnancy BMI was 25.54 (SD=4.10), placing most participants in the normal-weight to overweight range. Participants reported using the application on average 3.85 days per week (SD=1.91). The mean SUS score was 60.60 (SD=14.35). The most frequently reported emotional responses to the application were happiness (38.3%), curiosity (37.7%), and irritation (21.9%). Negative emotions such as sadness (5.6%), guilt (11.1%), insecurity (9.7%), and anger (2.8%) were reported less often. Feelings of being persuaded (15.9%) and surprised (8.2%) were also relatively low, while anxiety (0.5%) was scarcely reported.

Furthermore, Pearson correlations indicated that the variables of interest were significantly associated with each other (Supplemental Table 1, available online at https://www.mcpdigitalhealth.org/).

**Figure 2:**
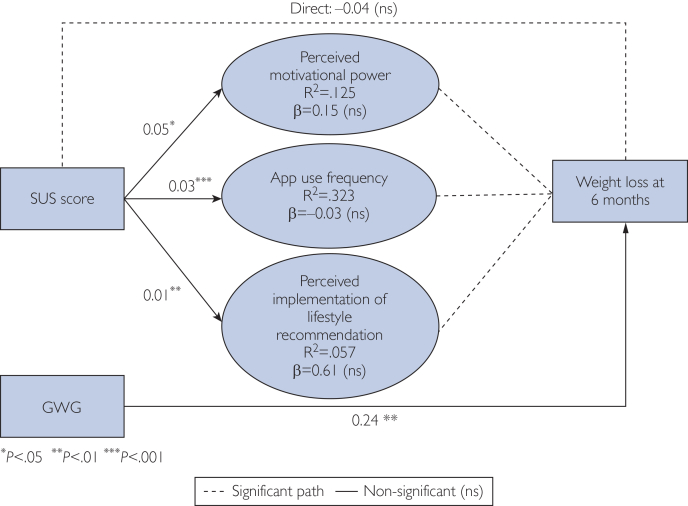
Path model of the associations between system usability scale score and weight loss at 6 months, with app use frequency, perceived motivational power, and perceived implementation of lifestyle recommendations as mediators. Values represent standardized regression coefficients (β) adjusted for maternal age, parity, prepregnancy body mass index, weight loss at 6 weeks postpartum, and gestational weight gain. Solid lines indicate significant paths; dashed lines indicate nonsignificant paths. ∗*P*<.05, ∗∗*P*<.01, ∗∗∗*P*<.001.

Mediation analysis: usability and weight loss at 6 months postpartum SUS scores were significantly associated with more frequent application use (b=0.05, *P*<.001), greater perceived motivational power (b=0.01, *P*<.036), and greater implementation of lifestyle recommendations (b=0.01, *P*=.036) (Figure 2 and Supplemental Table 2, available online at https://www.mcpdigitalhealth.org/). However, none of the 3 mediators were significantly associated with weight loss at 6 months postpartum. Among the covariates, only GWG was positively associated with weight loss at 6 months postpartum (b=0.24, *P*=.003).

**Figure 3:**
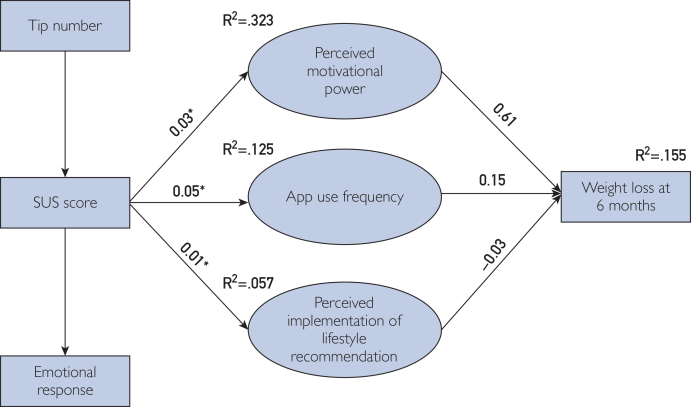
Path model of the associations between system usability scale score and weight loss at 6 months, with application use frequency, perceived motivational power, and perceived implementation of lifestyle recommendations as mediators. Values represent unstandardized regression coefficients adjusted for maternal age, parity, prepregnancy body mass index, weight loss at 6 weeks postpartum, and gestational weight gain. ∗*P*<.05, ∗∗*P*<.01, ∗∗∗*P* <.001.

Figure 3 and Supplemental Table 3 (available online at https://www.mcpdigitalhealth.org/) summarize the total, direct, and indirect effects. Neither the total effect (b=0.01, SE=0.02), nor the direct effect (b=−0.04, SE=0.02) of SUS score on weight loss at 6 months postpartum was significant. All indirect effects had confidence intervals that included zero, indicating no mediation.

### Usability and Emotional Responses

The multivariate model showed a significant association between usability and emotional responses, Pillai’s Trace=.221, F(10, 123)=2.49, *P*<.001, partial η^2^=.221.

Follow-up univariate tests showed that higher SUS scores were significantly associated with greater feelings of happiness (F(1,132)=10.85, *P*=.001, partial η^2^=.076), less irritation (F(1,132)=14.21, *P*<.001), partial η^2^=.097), greater curiosity (F(1,132)=6.89, *P*=.010, partial η^2^=.050), and a modest increase in feelings of being persuaded (F(1,132)=3.91, *P*=.050, partial η^2^=.029) (Table 2). No significant associations emerged for sadness, guilt, insecurity, surprise, anger or anxiety (all *P*>.20).

**Table 2:** Associations Between SUS Scores and Individual Emotional Responses

Emotional response	F(1,132)	*P*	Partial η^2^
Happiness	10.85	.001∗	.076
Irritation	14.20	<.001∗	.097
Curiosity	6.89	.010∗	.050
Persuaded	3.91	.050∗	.029
Sadness	0.28	.598	.002
Guilty	1.30	.256	.010
Insecurity	0.72	.397	.005
Surprised	1.55	.215	.012
Angry	0.20	.657	.001
Anxiety	0.83	.365	.006

Abbrevation: SUS, system usability scale.

∗ Significant *P* values.

## Discussion

This study examined the relationship between perceived application usability, perceived application use frequency, perceived application motivational power, perceived implementation of lifestyle recommendations, and weight loss at 6 months postpartum among women using the INTER-ACT application. On average, women lost 11.01 kg (SD=3.50) during the first 6 weeks postpartum and an additional 2.69 kg (SD=3.39) between 6 weeks at 6 months postpartum, resulting in a mean PPWR of 4.08 kg (SD=4.52). A substantial proportion (39%) showed EPPWR (≥5 kg), with some women even experiencing weight gain, underscoring the continued need for effective postpartum interventions to reduce PPWR.

In this context, evaluating the usability of the INTER-ACT application is crucial, as usability may influence adherence to lifestyle recommendations, perceived motivational power, and ultimately postpartum weight loss.

The mean SUS score of 60.60/100 suggests moderate usability, falling slightly below the commonly accepted benchmark of 70 for average usability.^34^, ^35^, ^36^ Although participants generally perceived the application as functional, these findings suggest there is potential to optimize the usability. Emotional responses were predominantly positive, with happiness and curiosity most frequently reported, while negative emotions such as sadness, anger, and anxiety were rarely experienced.

To further understand how these usability perceptions and affective responses relate to actual application behavior and adherence, correlation and mediation analyses were conducted. Perceived usability is considerably associated with perceived application use frequency, implementation of lifestyle recommendations, and motivational power. This aligns with prior literature suggesting that digital health tools with higher perceived usability promote sustained engagement and greater adherence.^37^ A well-designed, intuitive interface is a prerequisite for sustained engagement, as frustration or poor navigation can lead to disengagement regardless of potential health benefits.^38^^,^^39^ In addition, usability also shaped emotional responses: higher SUS scores were associated with greater happiness and curiosity and less irritation, suggesting that usability may act as both a technical and emotional determinant of UX.

Building on this, we also examined how the perceived balance of application content influenced usability ratings, highlighting another key factor for effective mHealth design.

The finding that perceiving the content as either too much or too little was associated with lower self-reported usability is particularly interesting for mHealth design. This supports a Goldilocks principle for digital health content: content should be neither overwhelming nor insufficient but calibrated so that users feel adequately supported without experiencing cognitive overload. Although this study did not directly test the role of content strategy or personalization, these factors could be important considerations for future mHealth design.^40^^,^^41^

In addition, this robust engagement pathway did not translate into significant weight loss. Despite clear associations between usability and engagement-related constructs, neither perceived application usability, perceived application use frequency, perceived motivational power, nor perceived implementation of lifestyle recommendations were significantly associated with weight loss at 6 months postpartum. This raises the question of whether participants overestimate their adherence or whether the behavioral changes achieved were simply insufficient to produce measurable health benefits.

The moderate usability level observed in this study may have further limited impact. Although a SUS score of 60.6 appears adequate to support basic use, it also indicates notable usability shortcomings. It is plausible that minor frictions, such as slow loading times, confusing menus, or suboptimal notifications, may have disrupted the integration of application-based strategies into daily routines and reduced the consistency needed for weight loss.

Next, the results emphasize that postpartum weight regulation is shaped by multiple powerful determinants that extend beyond the scope of a single application. The only variable significantly associated with PPWR in our model was GWG, a well-established factor in the literature.^26^ This indicated that strong biological and pre-existing behavioral patterns (eg, diet and exercise habits during pregnancy) may be more powerful determinants of postpartum weight than engagement with a postpartum mHealth tool. This raises the question of how much additional benefit can realistically be expected from lifestyle interventions initiated only after childbirth. Indeed, evidence from other interventions targeting PPWR suggests that, while some effects are observed, the overall impact is often modest and variable,^42^, ^43^, ^44^ highlighting the challenge of achieving substantial weight regulation through lifestyle changes alone. Unmeasured variables, such as sleep quality, stress, social support, mental health, and detailed dietary intake, likely contribute significantly as well.^1^^,^^45^^,^^46^

At the same time, our findings show that higher usability was significantly associated with specific emotional responses, which can influence how users perceive and interact with digital tools.^47^ Enhancing the usability of applications from moderate to good (SUS>70) can be achieved by minimizing sources of user irritation and customizing content delivery to better meet users’ emotional and practical needs, thereby improving overall satisfaction.^48^ Such strategies may also help address health equity concerns, while mobile applications provide accessible platforms for lifestyle support, their usability, emotional resonance, and perceived usefulness can differ widely across individuals, reflecting variations in digital health literacy, stress levels, or social support (50,51). Addressing these disparities in design and delivery will be essential to ensure that digital interventions equitably benefit diverse postpartum populations. This could be achieved through participation or co-creation, where end-users are involved in the design and testing process to ensure cultural, emotional, and practical relevance. Furthermore, tailoring the application’s content and features to individual needs, such as offering varying degrees of guidance, feedback, or emotional tone, may help accommodate differences in stress, motivation, or support systems. Given the heterogeneity of postpartum experiences, a single gold standard design is unlikely; instead, adaptable, user-centered solutions are likely more effective.

Taken together, these findings suggest that while usability and positive user experience are essential foundations for engagement, they do not guarantee clinical effectiveness. The postpartum period presents unique and overwhelming challenges that may overshadow the impact of digital tools alone. Future randomized controlled trials or longitudinal mixed-methods studies are needed to assess both short- and long-term outcomes while capturing user experience.

### Strengths and Limitations

A major strength of this study is the use of a theory-driven mediation model to examine complex behavioral pathways and the inclusion of a comprehensive set of factors, which provides a nuanced understanding of perceived effectiveness mechanisms in mHealth interventions. The multidimensional evaluation of usability goes beyond typical usability studies that often rely on single-item or ad hoc measures. An additional strength is the involvement of a multidisciplinary team, combining expertise in psychology, behavioral science, nutrition, and clinical practice, which enhances the study’s comprehensiveness and applicability.

Another strength is the integration of psychological constructs (perceived motivational power), behavioral indicators (implementation of lifestyle recommendations), and clinical outcomes (weight loss at 6 months postpartum). Furthermore, by focusing on intermediate behavioral mechanisms such as perceived motivational power and implementation, this study addresses the black box often left unexplored in digital intervention research. Rather than focusing solely on end outcomes or application usage frequency, it investigates how specific components of UX may influence meaningful health behaviors. Finally, the combination of self-reported psychological measures with objective outcome data (eg, weight change) strengthens the ecological validity of the findings.

Despite these strengths, several limitations must be acknowledged. First, the final analytic sample (N=138) has limited power to detect small to moderate indirect effects. High attrition, largely due to missing data and participant dropout, may have introduced bias. It is possible that women who completed the follow-up were more motivated, satisfied with the application, or engaged in their health, which could skew results and limit generalizability.

Second, the sample consisted exclusively of Dutch-speaking women who had experienced EGWG, which may limit the generalizability of the findings to more diverse populations or those with different perinatal profiles.

Another limitation is that prepregnancy weight was self-reported, whereas PPWR was objectively measured. As a result, PPWR was calculated using a combination of self-reported and objectively measured data and cannot be considered fully objective. The self-reported prepregnancy weight may be subject to recall bias, validity, and systematic underreporting, particularly given the substantial time interval (up to ∼40 weeks) between the actual prepregnancy weight and its reporting. This likely introduced measurement error in PPWR, which may have attenuated associations and contributed to the absence of statistically significant effects on this outcome. Also, the duration of the follow-up may have been insufficient to observe long-term effects of behavioral change on weight outcomes.

The last limitation is the use of self-reported application use frequency as a measure of engagement. Self-reported measures are subject to recall bias and social desirability bias, and participants may overestimate their actual application use. As a result, this measure may not accurately reflect true engagement behavior.

Future studies may benefit from mixed-method approaches that explore users’ subjective experiences in greater depth. To enhance real-time insight into user engagement and behavior, future research could benefit from integrating ecological momentary assessment or experience sampling methods, which would allow tracking of application use and emotional responses in daily life. Finally, the study focused on outcomes within the first 6 months postpartum. Although this is a crucial period for behavioral and physical adjustments, it may not fully capture the long-term sustainability of user engagement and weight-related changes. Furthermore, weight loss at 6 months postpartum was calculated as the difference between body weight at 6 weeks and 6 months postpartum, given that the intervention started at 6 weeks. As a result, the additional weight to be lost after 6 weeks may have been more limited, potentially reducing the need for or perceived benefit of the intervention. Consequently, women may have disengaged from the application, which could have contributed to slower weight loss during the later postpartum period.

## Conclusion

Although application usability is associated with perceived application use frequency, perceived motivational power, and perceived implementation of lifestyle recommendations, it is not associated with reduced weight loss at 6 months postpartum. Furthermore, usability also shapes emotional responses: higher usability scores were associated with considerably greater feelings of happiness and curiosity, and less irritation. However, these factors did not translate into significant weight loss. The strongest factor associated with PPWR remained GWG, highlighting that the profound biological and contextual challenges of pregnancy can outweigh the effect of digital support in the postpartum period. Future research should therefore not only aim to identify which usability and behavioral components enhance engagement after childbirth but also focus on strategies to optimize GWG during pregnancy to prevent postpartum weight retention.

## Potential Competing Interests

The authors report no competing interests.

## Ethics Statement

This study was approved by the Clinical Trial Center/Ethical Committee UZ Leuven (protocol code B322201730956/S59889).
