# Supplementary material for: Application Usability, Engagement, and Postpartum Weight Retention: Secondary Analysis of the INTER-ACT Randomized Controlled Trial
Source: Mayo Clin Proc Digit Health. 2026 Jun 9;4(3):100381. doi: 10.1016/j.mcpdig.2026.100381 (PMC13334793; doi:10.1016/j.mcpdig.2026.100381)
Supplement: Supplementary Material [file mmc1.docx]

**Supplemental Appendix**

**Table S1.** Pearson correlation matrix of weight loss, app usability, user experience, and emotional responses (N = 138).

| 1. Weight loss 6 months postpartum | — |  |  |  |  |  |  |  |  |  |  |  |  |  |  |  |  |  |  |  |  |  |
| --- | --- | --- | --- | --- | --- | --- | --- | --- | --- | --- | --- | --- | --- | --- | --- | --- | --- | --- | --- | --- | --- | --- |
| 2. PPWR | -.48** | — |  |  |  |  |  |  |  |  |  |  |  |  |  |  |  |  |  |  |  |  |
| 3. SUS score | -.02 | .05 | — |  |  |  |  |  |  |  |  |  |  |  |  |  |  |  |  |  |  |  |
| 4. Perceived motivational power | .11 | -.07 | .55** | — |  |  |  |  |  |  |  |  |  |  |  |  |  |  |  |  |  |  |
| 5. Implementation of lifestyle recommendations | .08 | .04 | .20* | .47** | — |  |  |  |  |  |  |  |  |  |  |  |  |  |  |  |  |  |
| 6. App use frequency | .07 | -.07 | .34** | .30** | .08 | — |  |  |  |  |  |  |  |  |  |  |  |  |  |  |  |  |
| 7. Too many tips | .03 | .00 | -.28* | -.19 | -.20 | -.05 | — |  |  |  |  |  |  |  |  |  |  |  |  |  |  |  |
| 8. Too little tips | -.08 | .14 | -.20* | -.18 | -.09 | .01 | .c | — |  |  |  |  |  |  |  |  |  |  |  |  |  |  |
| 9. Joy | .16 | -.06 | .27** | .33** | .38** | .27** | -.23* | -.17 | — |  |  |  |  |  |  |  |  |  |  |  |  |  |
| 10. Irritation | -.18* | .05 | -.32** | -.33** | -.29** | -.12 | .47** | .13 | -.22* | — |  |  |  |  |  |  |  |  |  |  |  |  |
| 11. Curiosity | .10 | -.03 | .21* | .15 | -.02 | -.13 | -.13 | -.10 | -.24** | -.37** | — |  |  |  |  |  |  |  |  |  |  |  |
| 12. Sadness | -.19* | .12 | -.06 | -.15 | -.06 | .02 | .11 | .05 | -.10 | .06 | -.17* | — |  |  |  |  |  |  |  |  |  |  |
| 13. Guilt | -.16 | .17* | .09 | -.05 | -.05 | -.07 | .02 | .06 | -.11 | -.01 | -.01 | .26** | — |  |  |  |  |  |  |  |  |  |
| 14. Insecurity | -.24** | .14 | -.08 | -.12 | -.05 | -.02 | .04 | .14 | -.12 | .07 | -.12 | .14 | .18* | — |  |  |  |  |  |  |  |  |
| 15. Surprise | .06 | .03 | .10 | .11 | .04 | .05 | .11 | .01 | .03 | -.09 | .04 | -.10 | .09 | .12 | — |  |  |  |  |  |  |  |
| 16. Anger | -.21* | .06 | -.05 | .03 | -.06 | .11 | .08 | -.15 | -.05 | .17 | -.04 | .03 | .32** | .23** | .10 | — |  |  |  |  |  |  |
| 17. persuaded | .15 | -.06 | .18* | .25** | .28** | .24** | -.09 | -.16 | .13 | -.15 | -.06 | -.06 | .02 | -.04 | .15 | .08 | — |  |  |  |  |  |
| 18. Anxiety | -.18* | -.03 | .06 | .06 | .05 | .07 | -.05 | -.05 | .12 | -.07 | .02 | .12 | .09 | .37** | .08 | .35** | .05 | — |  |  |  |  |
| 19. Weight loss 6 weeks postpartum | -.03 | -.37** | .18* | .15 | .01 | .08 | -.17 | -.16 | -.02 | -.07 | .19* | -.13 | -.08 | -.05 | -.17* | -.04 | -.03 | .16 | — |  |  |  |
| 20. EGWG | .11 | .41** | .11 | .07 | .09 | .05 | -.04 | -.06 | -.04 | -.09 | .20* | -.09 | .04 | -.11 | .05 | -.07 | -.01 | .05 | .27** | — |  |  |
| 21. BMI | -.19* | .09 | -.07 | -.09 | .01 | .02 | .22* | -.06 | -.06 | .10 | -.05 | .07 | .06 | -.00 | .19* | .09 | -.08 | .12 | -.29** | .30** | — |  |
| 22. Age | -.05 | -.11 | -.11 | .04 | -.01 | .06 | .12 | .07 | .04 | .02 | .07 | .12 | .06 | .07 | .02 | .09 | -.16 | .13 | -.10 | -.19* | .12 | — |
| 21. Parity | .12 | .04 | -.00 | .06 | .09 | .02 | -.02 | .15 | .08 | -.01 | .02 | -.07 | -.02 | -.03 | .06 | -.02 | -.04 | -.10 | -.25** | -.06 | .06 | .41** |

Note: *p < .05, **p < .01

**Table S2.** Mediation analyses of SUS Scores on weight loss at 6 months postpartum mediated through app use frequency, implementation of lifestyle recommendations, and perceived app motivational power.

|  | App Use Frequency (Days/Week) | | | Implementation of Lifestyle Recommendations | | | Perceived app motivational power | | | Weight loss at 6 Months | | |
| --- | --- | --- | --- | --- | --- | --- | --- | --- | --- | --- | --- | --- |
| Variable | Coeff | t | LLCI-ULCI | Coeff | t | LLCI-ULCI | Coeff | t | LLCI-ULCI | Coeff | t | LLCI/ULCI |
| Constant | -1.12 | -.52 | -5.35, 3.11 | 1.66 | 1.84 | -0.12, 3.44 | 0.40 | .50 | -1.17, 1.97 | 4.07 | 3.83 | -3.50, 11.64 |
| SUS score | **0.05***** | **4.10** | **-0.02, 0.07** | **0.01**** | **2.12** | **0.00, 0.02** | **0.03***** | **7.34** | **0.02, 0.04** | -0.04 | 0.02 | -0.09, 0.01 |
| Age | 0.05 | 1.02 | -0.04, 0.14 | -0.00 | -.08 | -0.04, 0.04 | 0.02 | 1.18 | -0.01, 0.05 | -0.04 | 0.08 | -0.21–0.12 |
| Parity | -0.02 | -.11 | -0.47, 0.42 | 0.09 | .96 | -0.10, 0.28 | 0.04 | .50 | -0.12, 0.21 | 0.51 | 0.40 | -0.28–1.30 |
| BMI | 0.02 | .51 | -0.06, 0.10 | 0.01 | .46 | -0.03, 0.28 | -0.01 | -.55 | -0.04, 0.02 | -0.13 | 0.07 | -0.27–-0.02 |
| Weight loss 6 weeks | 0.02 | .29 | -0.09, 0.12 | -0.01 | -.36 | -0.05, 0.04 | 0.01 | .52 | -0.03, 0.05 | -0.17 | 0.09 | -0.35, 0.02 |
| GWG | 0.00 | .10 | -0.08, 0.09 | 0.02 | 1.08 | -0.02, 0.06s | 0.01 | .52 | -0.02, 0.04 | **0.24**** | **0.08** | **0.08, 0.40** |
| App use frequency (days/week) |  |  |  |  |  |  |  |  |  | 0.15 | 0.16 | -0.16–0.46 |
| Implementation of lifestyle Recommendations |  |  |  |  |  |  |  |  |  | 0.61 | 0.47 | -0.32–1.54 |
| App motivation |  |  |  |  |  |  |  |  |  | -0.03 | 0.41 | -0.84–0.78 |
| Model | R² = .125, F(6,131) = 3.13** | | | R² = .323, F(6,131) = 10.43*** | | | R² = .057, F(6,131) = 1.32 | | | R² = .155, F(9,128) = 2.61** | | |

Note: *p < .05, **p < .01, ***p < .001.
LLCI, Lower Level Confidence Interval;
ULCI, Upper Level Confidence Interval.

**Table S3.** Overview of bootstrapped (5000 samples) standardized total, direct, and indirect effects.

| **Effect type** | **Effect (B)** | **SE / BootSE** | **95% CI** |
| --- | --- | --- | --- |
| **Total effect** | -0.01 | 0.02 | [-0.05, 0.02] |
| **Direct effect** | -0.04 | 0.02 | [-0.09, 0.01] |
| **Indirect effect (Total)** | 0.03 | 0.01 | [-0.00, 0.05] |
| – via App use frequency | 0.01 | 0.01 | [-0.01, 0.02] |
| – via perceived motivational power | 0.02 | 0.01 | [-0.01, 0.05] |
| – via Implementation of lifestyle recommendation | 0.00 | 0.00 | [-0.01, 0.01] |

**S4. System Usability Score (SUS) questionnaire.**

1. I think that I would like to use this system frequently.
2. I found the system unnecessarily complex.
3. I thought the system was easy to use.
4. I think that I would need the support of a technical person to be able to use this system.
5. I found the various functions in this system were well integrated.
6. I thought there was too much inconsistency in this system.
7. I would imagine that most people would learn to use this system very quickly.
8. I found the system very cumbersome to use.
9. I felt very confident using the system.
10. I needed to learn a lot of things before I could get going with this system.
